# The stimulation of mitogenic signaling pathways by N-POMC peptides

**DOI:** 10.1016/j.mce.2008.09.021

**Published:** 2009-03-05

**Authors:** David J. Pepper, Andrew B. Bicknell

**Affiliations:** School of Biological Sciences, The University of Reading, Whiteknights, PO Box 228, Reading, Berkshire RG6 6AJ, UK

**Keywords:** Adrenal, POMC, N-POMC, ERK, AKT, Receptor tyrosine kinase

## Abstract

The N-terminal fragment of pro-opiomelancortin (POMC) has been shown previously to act as an adrenal mitogen. However, little is known about the molecular mechanisms by which mitogenesis is stimulated, although it has been shown that N-POMC_1–28_ stimulates the ERK pathway in human H295R cells. We have investigated signaling stimulated by N-POMC_1–28_ and N-POMC_1–49_ in the mouse Y1 cell line and found that both peptides stimulate ERK phosphorylation with maximal stimulation being achieved within 5 min. Similar results were observed for both MEK and c-Raf phosphorylation, although N-POMC_1–49_ stimulated the phosphorylation of Akt more robustly than N-POMC_1–28_.

We also investigated the expression of tyrosine kinase receptors in adrenal cells. PCR utilizing degenerate primers was performed on cDNA from both Y1 cells and rat adrenal tissue. Sequencing of 114 clones from each cDNA population revealed the expression of a number of receptors, several of which have not been described previously in the adrenal.

## 1 Introduction

The cortex of the mammalian adrenal gland is subdivided into three concentric zones that are both functionally and morphologically distinct. The outer zona glomerulosa produces mineralocorticoids while the inner zona fasciculata and zona reticularis synthesize glucocorticoids (McNicol, 1992). It has long been appreciated that the cells comprising these three zones are constantly renewed allowing for the size of each zone (and hence their steroidogenic potential) to be constantly moderated (Chester-Jones, 1976).

It is believed that all the cells in the cortex are derived from a narrow band of stem cells located at the periphery of the gland and the daughter cells migrate towards the centre of the gland phenotypically switching into the various functional zones before undergoing apoptosis at the zona reticularis/medulla boundary (Estivariz et al., 1992). This proliferation is dependent upon peptides derived from the ACTH precursor pro-opiomelanocortin (POMC) since surgical removal of the pituitary (Deane and Greep, 1946) or suppression of corticotroph activity using dexamethasone (Bransome, 1968; Wright et al., 1974) (which results in down regulation of all POMC peptides) results in adrenal atrophy. This data has also been further substantiated by targeted disruption of the POMC gene where the adrenal rapidly atrophies soon after birth (Yaswen et al., 1999; Karpac et al., 2005).

The identity of the POMC-derived mitogen has been historically controversial. Many people have suggested that ACTH is the main stimulus of adrenal growth. Indeed, in super physiological doses it does promote an increase in adrenal size, although mainly by stimulating cellular hypertrophy. Conversely, studies *in vitro* have suggested that ACTH is anti-mitogenic to both primary adrenal cells (Ramachandran and Suyama, 1975) and the Y1 mouse adrenocortical cell line (Masui and Garren, 1971). The underlying cause of this apparent paradox is thought to be the result of the increase in cAMP levels stimulated by ACTH, since Y1 cells harboring dominant inhibitory mutations in protein kinase A are resistant to the growth-inhibitory effects of ACTH (Olson et al., 1993). Other experiments have suggested also that ACTH may not be the sole mitogenic drive to the adrenal. *In vivo* immuno-neutralization with anti-ACTH antiserum, although decreasing steroid output, actually results in an increase in adrenal size (Rao et al., 1978; Estivariz et al., 1982); the decrease in steroid levels resulting in an up-regulation of not only ACTH, but also all POMC peptides including any other mitogenic peptide. These observations encouraged our lab to investigate a role in adrenal cell proliferation for the N-terminal fragment of POMC, alternatively known as the 16K fragment, N-POMC or pro-γ-MSH (Estivariz et al., 1982; Lowry et al., 1983); a 76 residue (74 in rodents) glycopeptide that is co-secreted with ACTH from the pituitary into the circulation. Initial observations showed that the peptide had no significant mitogenic activity towards the adrenal. However, two shorter peptides (both of which were extraction artifacts) purified from human pituitaries, namely N-POMC_1–28_ and N-POMC_2–54_ were found to be potent mitogens *in vitro* (Estivariz et al., 1982). Since neither of these peptides are products of normal pituitary processing, it was suggested that the mitogenic fragment was generated by cleavage of pro-γ-MSH following secretion from the pituitary. This idea was supported in a series of elegant immuno-neutralization experiments showing that the compensatory growth response following unilateral adrenalectomy could be blocked using an antibody against γ-MSH; the explanation being that the antibody blocked the 49/50 dibasic cleavage site found in pro-γ-MSH (Lowry et al., 1983). In 2001 we identified the enzyme that performs this cleavage, which we named adrenal secretory protease (AsP) (Bicknell et al., 2001). Initial characterization of AsP with synthetic substrates suggested that the adrenal mitogen was N-POMC_1–52_ although our more recent work with the natural peptide substrate suggests cleavage is more likely to occur at the dibasic site, resulting in the generation of N-POMC_1–49_ (unpublished observations).

Only one study has investigated how N-POMC stimulates adrenal proliferation at the molecular level. Fassnacht et al. (2003) showed that synthetic N-POMC_1–28_ stimulated proliferation of human H295R cells and the peptide activated the ERK pathway.

The aim of this study was to extend this work and examine the signaling pathways stimulated in the Y1 adrenocortical cell line. It also aimed to survey the expression of receptor tyrosine kinase molecules in an attempt to identify a possible candidate by which N-POMC could stimulate adrenal cell proliferation.

## 2 Experimental procedures

### 2.1 Source of peptides

N-POMC_1–49_ was purified to homogeneity from bovine pituitary tissue (Pel-Freez Biologicals, Rogers, AZ, USA) using ion exchange and reversed phase high-performance liquid chromatography (RP-HPLC) as previously described (Bennett, 1986a) and quantified using amino acid analysis. N-POMC_1–28_ was custom synthesized by Bachem (St Helens, UK).

### 2.2 Cell culture

Mouse Y1 adrenal cortical tumour cells were obtained from the European Collection of Animal Cell Cultures (ECACC). These cells have been used by us previously (Bicknell et al., 2001) and now do not express any of the known melanocortin receptors. Cells were maintained in a humidified incubator at 37 °C with 5% CO_2_ in growth media consisting of Hams F12 media (Sigma, Poole, Dorset, UK) supplemented with 10% fetal bovine serum (Invitrogen, Paisley, Scotland), 2 mM l-glutamine (Invitrogen, Paisley, Scotland) and 100 μg/ml streptomycin and 100 U/ml penicillin G (Invitrogen, Paisley, Scotland).

### 2.3 Western blot analysis

Y1 cells were grown to confluence in 90-mm dishes and serum starved for 48 h in Hams F12 supplemented with 2 mM glutamine and 100 μg/ml streptomycin and 100 U/ml penicillin G.

Serum starved cells were treated with 1 nM of either N-POMC_1–49_ or N-POMC_1–28_ given in the same media and incubated for various times. As an additional control cells were stimulated with just media alone for each time point to confirm that any effect was due solely to the addition of the peptide (results not shown). After incubation, protein was harvested by lysing the cells directly in 500 μl of 1x reducing sample treatment buffer (Laemmli, 1970). After brief sonication, and heating to 94 °C for 5 min, 10 μl of each sample was separated on a or 10% SDS-PAGE gel and transferred to PVDF membrane (Bio-Rad, Hemel Hempstead, UK). The membrane was blocked by incubating with Tris-buffered saline (TBS—10 mM Tris HCL (pH 7.4), 150 mM sodium chloride) containing 5% skimmed milk powder and 0.1% Tween 20 (Sigma, Poole, UK) for 2 h at room temperature. After blocking, the membranes were probed with either a total ERK1/2 antibody, or one of the phospho specific antibodies: ERK1/2 (Thr 202/Tyr 204), c-RAF (Ser 338), MEK1/2 (Ser 217/221) or Akt (Ser 473) all at a 1:2000 dilution (Cell Signaling Technology (NEB), Hitchin, UK). All primary antibodies were then detected using a horse radish peroxidase (HRP)-conjugated secondary goat anti-rabbit antibody (Dako, Ely, UK) and visualised using an enhanced chemiluminescence (ECL) system, as per manufacturer's instructions (Amersham, Buckinghamshire, UK). All blots were carried out on three independent samples.

After immunoblotting, bands were analyzed by densitometry using a GS-710 calibrated imaging densitometer (Bio-Rad, Hemel Hempstead, UK).

### 2.4 Amplification of receptor tyrosine kinase (RTK) sequences

To analyze for RTK expression the polymerase chain reaction (PCR) was used essentially as previously described (Tanaka et al., 1998). Briefly, degenerate oligonucleotide primers (forward 5′ CAYMGIGAYYTIGCIGCIMGIAA 3′ reverse 5′ AYICCRWAISWCCAIACRTC 3′) were synthesized (Sigma-Genosys, Cambridge, UK) and used to set up PCR reactions containing cDNA synthesized from either whole rat adrenal gland tissue or from Y1 cells. Reactions were initially denatured for 1 min at 94 °C and then subjected to 35 cycles of 94 °C (20 s), 45 °C (20 s), 72 °C (1 min) before being analyzed by agarose gel electrophoresis. Bands of interest were excised, purified using a Qiagen gel extraction kit (Qiagen, Crawley, UK) and then T/A cloned into pGEM-T (Promega, Southampton, UK). Clones were subsequently grown up, plasmid isolated (Qiagen, Crawley, UK) and inserts sequenced (GATC Biotech, Konstanz, Germany).

### 2.5 Statistical analysis

Statistical analysis was performed using one-way analysis of variance (ANOVA) and the Tukey–Kramer post-analysis test. Results were considered statistically significant if the *p*-value was <0.05. Statistical analysis was performed using Graphpad Prism (Graphpad Software, San Diego, USA).

## 3 Results

### 3.1 Both N-POMC_1–28_ and N-POMC_1–49_ activate the ERK signaling pathway

**Fig. 1:**
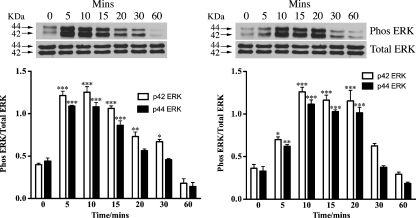
Serum starved Y1 cells were stimulated with either 1 nM of N-POMC_1–28_ (A) or N-POMC_1–49_ (B) for various times. Cell lysates were analyzed by immunoblotting with antibodies against total p44/42 ERK and phospho p44/42 ERK. Band intensities are normalized to those of the total amount of either p44 or p42 ERK and expressed as the mean (±S.E.M.) of three independent experiments. *p < 0.05, **p < 0.01, ***p < 0.001 relative to value at 0 min.

Since synthetic N-POMC_1–28_ has been shown previously to activate p44/42 ERK (Fassnacht et al., 2003) we were interested to see if this peptide could also activate this signaling pathway in the commonly used mouse Y1 adrenocortical cell line. We were also interested to investigate if the longer N-POMC_1–49_ peptide (which more likely represents the natural product of AsP) could also activate this pathway and in a similar manner to N-POMC_1–28_. Y1 cells were serum starved for 48 h to minimize the high levels of endogenous ERK phosphorylation present in these cells before being stimulated with 1 nM of each peptide. Cells were consequently harvested at 0, 5, 10, 15, 20, 30 and 60 min and immunoblotted for both total p44/42 ERK and phospho p44/42 ERK. Both peptides were found to strongly stimulate the phosphorylation of both p44 and p42 ERK (Fig. 1) with the response of N-POMC_1–49_ being slightly slower than that observed for N-POMC_1–28_.

### 3.2 Both N-POMC_1–28_ and N-POMC_1–49_ activate upstream regulators of p44/42 ERK

**Fig. 2:**
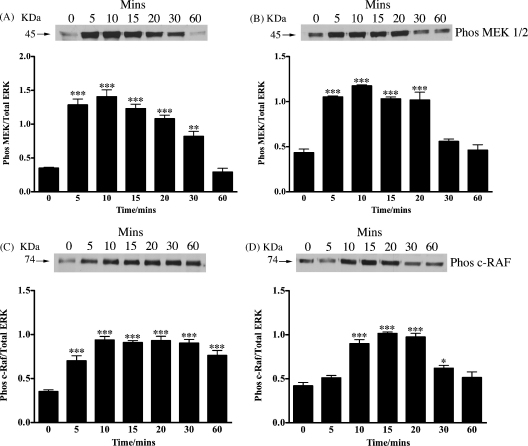
Serum starved Y1 cells were stimulated with either 1 nM of N-POMC_1–28_ (A and C) or N-POMC_1–49_ (B and D) for various times. Cell lysates were analyzed by immunoblotting with antibodies against phospho MEK1/2 (A and B) and phospho c-RAF (C and D) ERK. Band intensities are normalized to those of the total amount of p44 ERK and expressed as the mean (±S.E.M.) of three independent experiments. *p < 0.05, **p < 0.01, ***p < 0.001 relative to value at 0 min.

**Fig. 3:**
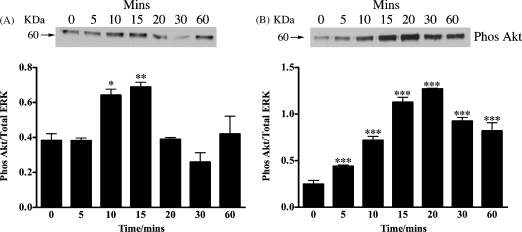
Serum starved Y1 cells were stimulated with either 1 nM of N-POMC_1–28_ (A) or N-POMC_1–49_ (B) for various times. Cell lysates were analyzed by immunoblotting with antibodies against phospho Akt. Band intensities are normalized to those of the total amount of p44 ERK and expressed as the mean (±S.E.M.) of three independent experiments. *p < 0.05, **p < 0.01, ***p < 0.001 relative to value at 0 min.

As both peptides could clearly stimulate the phosphorylation of ERK we next investigated if they could also phosphorylate the upstream regulators of ERK: MEK and c-RAF. Using immunoblotting together with specific phospho antibodies, both peptides were found to stimulate the phosphorylation of both MEK and c-RAF (Fig. 2). We also investigated if N-POMC_1–28_ and N-POMC_1–49_ were capable of stimulating the phosphorylation of Akt, a key molecule in the PI3 kinase pathway. N-POMC_1–49_ was found to stimulate a robust phosphorylation of Akt, while in contrast the response to N-POMC_1–28_ was weaker (Fig. 3).

### 3.3 Adrenal cells express numerous tyrosine kinase molecules

**Fig. 4:**
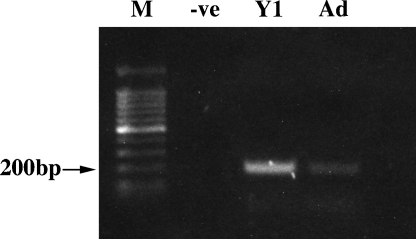
PCR using degenerate oligonucleotides of genes containing the tyrosine kinase catalytic domain in Y1 cells and also in normal rat adrenal gland. The expected band size of approx 200 bp was excised, cloned and 114 clones of each sequenced.

The identity of the receptor for N-POMC remains elusive. In an attempt to isolate possible candidate molecule we used PCR with degenerate oligonucleotide primers to amplify the conserved sequence within the catalytic domains of receptor-type tyrosine kinase molecule as has been described previously (Tanaka et al., 1998). Using this approach with cDNA derived from both Y1 cells and also from rat adrenal tissue we successfully amplified a band of the expected 200 bp (Fig. 4). The bands were excised and cloned into pGEM-T and colonies picked for plasmid isolation and subsequent sequencing. One hundred and fourteen clones from each cDNA were successfully sequenced. Every clone represented a tyrosine kinase molecule, although many of them were found not to be receptors (Table 1).

## 4 Discussion

Although there is a substantial amount of evidence supporting the role of the N-terminal POMC peptides in the regulation of adrenal growth, little is known about the receptor that they work through or the underlying signaling mechanisms. In a study by Fassnacht et al. (2003) it was shown that synthetic N-POMC_1–28_ could promote the growth of both human H295R cells and bovine primary adrenal cells. They also showed that this peptide could stimulate the phosphorylation of p44/42 ERK but not c-Jun or p38.

Encouraged by this study, we decided to investigate the effects of N-POMC on the commonly used mouse Y1 adrenal cell line. There have been a few studies using this cell line that have shown that ACTH can rapidly phosphorylate both p44/42 ERK via a cAMP independent manner (Lotfi et al., 1997; Le and Schimmer, 2001). However, prolonged treatment with ACTH does not stimulate division of the cells, although a short transient stimulation of G_1_-arrested cells with ACTH does result in an increase in cell division. The Y1 cells used in this study were obtained from the ECACC and do not express either the melanocortin 2 receptor (MC2R) or the melanocortin 5 receptor (MC5R) and are consequently unresponsive to ACTH. We choose to use two N-POMC peptides, synthetic N-POMC_1–28_ and N-POMC_1–49_. We used N-POMC_1–28_ since it was used by Fassnacht et al. and has also been shown previously to stimulate thymidine incorporation in primary rat adrenal cells (Estivariz et al., 1982). We used also purified bovine N-POMC_1–49_ which is a processed product from the intermediate lobe. This peptide more likely represents the natural mitogenic fragment, although it lacks the O-linked glycan found on Thr45 since the PC2 mediated cleavage in the pituitary of the 49/50 dibasic site is blocked by the presence of this glycan (Bennett, 1986b). Whether the glycan is necessary for biological activity is currently unclear. Both peptides were given at a dose of 1 nM, a dose that was estimated to be sufficient to give a robust response.

In agreement with Fassnacht et al. both peptides were found to stimulate a rapid increase in the phosphorylation of both p44 and p42 ERK, within 10 min of application that gradually decreased, returning to basal within 1 h. In a similar manner MEK1/2, the kinase that mediates the phosphorylation of ERK and c-RAF, a kinase that phosphorylates MEK, were also robustly phosphorylated in a similar pattern to that observed for ERK. Interestingly, N-POMC_1–49_ appears to phosphorylate both MEK1/2 and p44/42 ERK in a slightly slower manner than N-POMC_1–28_. This observation led us to investigate the effect of the peptides on the phosphorylation of Akt, a central molecule in the PI3 kinase pathway. The constitutively high levels of c-Ki-Ras-GTP observed in Y1 cells have been reported to result in high basal level of Akt phosphorylation (Forti et al., 2002). However, since Akt has been shown previously to phosphorylate and deactivate c-RAF with the effect of reducing the activation of the ERK pathway (Rommel et al., 1999) we hypothesised that there may be differing effect of the two N-POMC peptides on the PI3 kinase pathway. Our results showed that N-POMC_1–49_ produced a far more robust phosphorylation than N-POMC_1–28_ and may explain the difference in the observed pattern of MEK1/2 and p44/42 ERK phosphorylation. An alternative explanation is that the molecules involved in ERK de-phosphorylation (e.g. ERK phosphatases) are also differentially activated.

These observations warrant further investigation since it suggests that the way that the two peptides stimulate intracellular signaling pathways are not identical.

Although N-POMC peptides robustly stimulate ERK phosphorylation, it is not clear if this is sufficient to lead to cell division. It may be possible that, in a similar manner to ACTH, that prolonged stimulation results in an inhibition of growth (Lotfi et al., 1997). Further studies are required to investigate if this is the case.

In an attempt to investigate a potential receptor for N-POMC, we used PCR with degenerate oligonucleotides designed around the catalytic domains of receptor type tyrosine kinase molecules. We reasoned that the receptor would be likely to be a member of this family, supported by the observation that both peptides stimulate a clear increase in total tyrosine phosphorylation by Western blot (unpublished observations). This approach has been used successfully before in the thyroid (Tanaka et al., 1998) and in our hands resulted in the amplification of an approx 200 bp fragment from Y1 cells and also from rat adrenal tissue. These bands were cloned and 114 clones of each subsequently sequenced. Every clone contained a tyrosine kinase domain, although many were not receptors but intracellular signaling molecules. Unsurprisingly, the profile of expressed genes was different between the Y1 cells and adrenal tissue. The frequencies of each cDNA are summarized in Table 1. Despite there being no obvious candidates for an N-POMC receptor, the results are still of interest although the functional significance of some of the receptors in the adrenal remains to be determined. The most abundant receptor expressed appears to be insulin-like growth factor 1 receptor (IGF1-R): a receptor known to be expressed in the adrenal. The Y1 cells also expressed Axl, a receptor that has transforming activity in NIH-3T3 cells (O’Bryan et al., 1991; Liu and Yamane, 1995). Its ligand, known as Gas6, promotes mitogenesis and is generally thought to act in an autocrine manner (Varnum et al., 1995; Goruppi et al., 1996). Another interesting receptor whose expression was observed solely in Y1 cells was Ryk, a receptor involved with the Wnt signaling pathway (Lu et al., 2004) and interestingly has no catalytic activity (Hovens et al., 1992; Stacker et al., 1993). This receptor has been implicated in many aspects of tissue development. Both Y1 cells and adrenal tissue express the fibroblast-growth factor receptor (FGF-R), which is not unexpected since both tissues, and especially Y1 cells, are responsive to FGF (Gospodarowicz and Handley, 1975; Gospodarowicz et al., 1977).

Single occurrences of several receptors were found in the adrenal gland including hepatocyte growth factor receptor and vascular endothelial growth factor (VEGF) receptor. The single occurrences of several genes begs the question as to whether enough clones were sequenced to identify all the genes present in the PCR product and in the future a micro-array approach would possibly provide a more complete dataset.

In summary we have shown that that N-POMC peptides can stimulate the ERK signaling pathway not only in the human H295R cell lines but also in mouse Y1 cells. We have also shown that Y1 cells and adrenal tissue express a number of tyrosine kinase receptors, although the full functional significance of these is unclear and warrants possible further investigation.

## Figures and Tables

**Table 1:** List of tyrosine kinase genes and the frequency they were identified isolated from Y1 cells and rat adrenal tissue.

Gene name	Type of tyrosine kinase	Number of clones in Y1 cells	Number of clones in rat adrenal gland
IGF1-R	Receptor	57	24
Jak 1	Signaling molecule	23	28
Ryk	Receptor	9	0
Axl	Receptor	11	2
FGF-R	Receptor	2	3
Tyk2	Signaling molecule	3	31
Megakaryocyte associated tyrosine kinase	Signaling molecule	2	0
c-abl	Signaling molecule	3	2
Jak 3	Signaling molecule	2	6
SRC	Signaling molecule	1	5
FER	Signaling molecule	1	5
Jak 2	Signaling molecule	0	3
CSF-1 R	Receptor	0	1
VEGF-R	Receptor	0	1
HGF-R	Receptor	0	1
v-ras	Signaling molecule	0	1
Unknown	?	0	1

Total		114	114

Summaries of the frequencies of tyrosine kinase domain cDNAs isolated from Y1 cells and normal rat adrenal gland. IGF1-R: insulin-like growth factor 1 receptor, FGF-R: fibroblast growth factor receptor, CSF-1R: colony stimulating factor 1 receptor, VEGF-R: vascular endothelial growth factor receptor, HGF-R: hepatocyte growth factor receptor.

## References

[bib1] Bennett H.P.J. (1986). Use of ion-exchange Sep-Pak cartridges in the batch fractionation of pituitary peptides. J. Chromatogr..

[bib2] Bennett H.P.J. (1986). Biosynthetic fate of the amino-terminal fragment of pro-opiomelanocortin within the intermediate lobe of the mouse pituitary. Peptides.

[bib3] Bicknell A.B., Lomthaisong K., Woods R.J., Hutchinson E.G., Bennett H.P.J., Gladwell R.T., Lowry P.J. (2001). Characterization of a serine protease that cleaves Pro-Gamma-Melanotropin at the adrenal to stimulate growth. Cell.

[bib4] Bransome E.D. (1968). Regulation of adrenal growth. Differences in the effects of ACTH in normal and dexamethasone-suppressed guinea pigs. Endocrinology.

[bib5] Chester-Jones I.J. (1976). Evolutionary aspects of the adrenal cortex and its homologues. J. Endocrinol..

[bib6] Deane H.W., Greep R.O. (1946). Morphological and histochemical study of rat's adrenal cortex after hypophysectomy with comments on liver. Am. J. Anat..

[bib7] Estivariz F.E., Iturriza F., McLean C., Hope J., Lowry P.J. (1982). Stimulation of adrenal mitogenesis by N-terminal proopiocortin peptides. Nature.

[bib8] Estivariz F.E., Lowry P.J., Jackson S., James V.H.T. (1992). Control of adrenal growth. The Adrenal Gland.

[bib9] Fassnacht M., Hahner S., Hansen I.A., Kreutzberger T., Zink M., Adermann K., Jakob F., Troppmair J., Allolio B. (2003). N-terminal proopiomelanocortin acts as a mitogen in adrenocortical tumor cells and decreases adrenal steroidogenesis. J. Clin. Endocrinol. Metab..

[bib10] Forti F.L., Schwindt T.T., Moraes M.S., Eichler C.B., Armelin H.A. (2002). ACTH promotion of p27(Kip1) induction in mouse Y1 adrenocortical tumor cells is dependent on both PKA activation and Akt/PKB inactivation. Biochemistry.

[bib11] Goruppi S., Ruaro E., Schneider C. (1996). Gas6, the ligand of Axl tyrosine kinase receptor, has mitogenic and survival activities for serum starved NIH3T3 fibroblasts. Oncogene.

[bib12] Gospodarowicz D., Handley H.H. (1975). Stimulation of division of Y1 adrenal cells by a growth factor isolated from bovine pituitary glands. Endocrinology.

[bib13] Gospodarowicz D., Ill C.R., Hornsby P.J., Gill G.N. (1977). Control of bovine adrenal cortical cell proliferation by fibroblast growth factor. Lack of effect of epidermal growth factor. Endocrinology.

[bib14] Hovens C.M., Stacker S.A., Andres A.C., Harpur A.G., Ziemiecki A., Wilks A.F. (1992). RYK, a receptor tyrosine kinase-related molecule with unusual kinase domain motifs. Proc. Natl. Acad. Sci. U.S.A..

[bib15] Karpac J., Ostwald D., Bui S., Hunnewell P., Shankar M., Hochgeschwender U. (2005). Development, maintenance, and function of the adrenal gland in early postnatal proopiomelanocortin-null mutant mice. Endocrinology.

[bib16] Laemmli U.K. (1970). Cleavage of structural proteins during the assembly of the head of bacteriophage T4. Nature.

[bib17] Le T., Schimmer B.P. (2001). The regulation of MAPKs in Y1 mouse adrenocortical tumor cells. Endocrinology.

[bib18] Liu E.T., Yamane H.K. (1995). Axl receptor tyrosine kinase stimulated by the vitamin K-dependent protein encoded by growth-arrest-specific gene 6. Nature.

[bib19] Lotfi C.F.P., Todorovic Z., Armelin H.A., Schimmer B.P. (1997). Unmasking a growth-promoting effect of the adrenocorticotrophic hormone in Y1 mouse adrenocortical tumor cells. J. Biol. Chem..

[bib20] Lowry P.J., Silas L., McLean C., Linton E.A., Estivariz F.E. (1983). Pro-gamma-melanocyte-stimulating hormone cleavage in adrenal gland undergoing compensatory growth. Nature.

[bib21] Lu W., Yamamoto V., Ortega B., Baltimore D. (2004). Mammalian Ryk is a Wnt coreceptor required for stimulation of neurite outgrowth. Cell.

[bib22] Masui H., Garren L.D. (1971). Inhibition of replication in functional mouse adrenal tumor cells by adrenocorticotropic hormone mediated by adenosine 3′:5′-cyclic monophosphate. Proc. Natl. Acad. Sci. U.S.A..

[bib23] McNicol A.M., James V.H.T. (1992). The human adrenal gland: aspects of structure, function and pathology. The Adrenal Gland.

[bib24] O’Bryan J.P., Frye R.A., Cogswell P.C., Neubauer A., Kitch B., Prokop C., Espinosa R., Le Beau M.M., Earp H.S., Liu E.T. (1991). Axl, a transforming gene isolated from primary human myeloid leukemia cells, encodes a novel receptor tyrosine kinase. Mol. Cell. Biol..

[bib25] Olson M.F., Krolczyk A.J., Gorman K.B., Steinberg R.A., Schimmer B.P. (1993). Molecular basis for the 3′,5′-cyclic adenosine monophosphate resistance of Kin mutant Y1 adrenocortical tumor cells. Mol. Endcocrinol..

[bib26] Ramachandran J., Suyama A.T. (1975). Inhibition of replication of normal adrenocortical cells in culture by adrenocorticotropin. Proc. Natl. Acad. Sci. U.S.A..

[bib27] Rao A.J., Long J.A., Ramachandran J. (1978). Effects of antiserum to adrenocorticotropin on adrenal growth and function. Endocrinology.

[bib28] Rommel C., Clarke B.A., Zimmermann S., Nuñez L., Rossman R., Reid K., Moelling K., Yancopoulos G.D., Glass D.J. (1999). Differentiation stage-specific inhibition of the Raf-MEK-ERK pathway by Akt. Science.

[bib29] Stacker S.A., Hovens C.M., Vitali A., Pritchard M.A., Baker E., Sutherland G.R., Wilks A.F. (1993). Molecular cloning and chromosomal localisation of the human homologue of a receptor related to tyrosine kinases (RYK). Oncogene.

[bib30] Tanaka K., Nagayama Y., Nakano T., Takamura N., Namba H., Fukada S., Kuma K., Yamashita S., Niwa M. (1998). Expression profile of receptor-type protein tyrosine kinase genes in the human thyroid. Endocrinology.

[bib31] Varnum B.C., Young C., Elliott G., Garcia A., Bartley T.D., Fridell Y.W., Hunt R.W., Trail G., Clogston C., Toso R.J., Yanagihara D., Bennett L., Sylber M., Merewether L.A., Tseng A., Escobar E., Liu E.T., Yamane H.K. (1995). Axl receptor tyrosine kinase stimulated by the vitamin K-dependent protein encoded by growth-arrest-specific gene 6. Nature.

[bib32] Wright N.A., Appleton D.R., Morley A.R. (1974). Effect of dexamethasone on cell population kinetics in the adrenal cortex of the prepubertal male rat. J. Endocrinol..

[bib33] Yaswen L., Diehl N., Brennan M.B., Hochgeschwender U. (1999). Obesity in the mouse model of pro-opiomelanocortin deficiency responds to peripheral melanocortin. Nat. Med..

